# Bringing Back the Balance: Domain-General Processes Are Also Important in Numerical Cognition

**DOI:** 10.3389/fpsyg.2017.00499

**Published:** 2017-03-30

**Authors:** Mateusz Hohol, Krzysztof Cipora, Klaus Willmes, Hans-Christoph Nuerk

**Affiliations:** ^1^Institute of Philosophy and Sociology of the Polish Academy of SciencesWarsaw, Poland; ^2^Copernicus Center for Interdisciplinary StudiesCracow, Poland; ^3^Department of Psychology, University of TuebingenTuebingen, Germany; ^4^Department of Neurology, University Clinic Rheinisch-Westfälische Technische Hochschule Aachen UniversityAachen, Germany; ^5^Leibnitz-Institut für WiessenmedienTuebingen, Germany; ^6^LEAD Graduate School and Research NetworkTuebingen, Germany

**Keywords:** numerical cognition, number processing, domain-general processes, cognitive control, inhibition, visual grouping, allocation of attention

In the field of numerical cognition it is often highlighted that the domain-specific systems, referred to as “Approximate Number System” (ANS), or “The Number Sense” (NS)^1^, constitute the basis for mathematical skills (Feigenson et al., 2004; Dehaene, 2011). However, recently, Leibovich et al. (2016) stressed the role of domain-general factors, especially cognitive control, in the formation of elementary numerical abilities. Here we would like to show that these domain-general processes are not only crucial for the formation of number concepts, but in our view, appear to be integral to all aspects of number processing.

Performing both simple numerical processing tasks as well as advanced mathematics requires involvement of domain-general processes apart from domain-specific components. The input material has to be perceived and preprocessed, if necessary stored in (working) memory, processed, and a decision needs to be made. Eventually, all this may lead to a response being given. For the importance of such processes see e.g., LeFevre et al. (2005) and Rousselle et al. (2004). Nevertheless, as numerous studies have shown, domain-general processes cannot be considered only to be providing material to domain-specific processes and then sending back the results. The domain-general processes are rather deeply involved in the information processing path. Focusing on domain-general processes seems necessary from an empirical point of view as well. Over two decades of extensive research aimed to show a relationship between signatures of elementary number/quantity processing^2^ and math skills have not led to consistent results (De Smedt et al., 2013; Lyons et al., 2015). Observed weak relationships between ANS measures and math achievement (r's ≈ 0.15) often vanish altogether when domain general factors are controlled for (e.g., Gilmore et al., 2013; Szucs et al., 2014). Importantly, not only performance in non-symbolic tasks does not seem to be genuinely related to arithmetic skills. The same holds true also for Spatial-Numerical Associations (SNA) and signatures of elementary numerical processing (Cipora and Nuerk, 2013; Cipora et al., 2015, 2016). While any inference from elementary non-symbolic numerical cognition to advanced arithmetic capabilities (the symbol grounding problem; see Leibovich and Ansari, 2016 for review) is far from being clear-cut, there is no doubt that domain-general factors play an important role, both in the case of tasks that are used for measuring NS/ANS (Szucs et al., 2014), and in more complex math performance (Desoete, 2015).

To provide an overview, in the following paragraphs we present three examples that cover domain-general factors operating at varying levels of information processing: from visual grouping through strategic attentional allocation up to inhibition. We discuss how these domain-general processes influence varied areas of numerical cognition, starting from dot set comparisons/estimations through multi-digit number processing up to calculation. These examples are not exhaustive but are aimed at showing the tight relationship between domain-general cognitive factors and number processing at different levels.

## Visual grouping

Despite spatial arrangement of digits being irrelevant to the operation order from a content point of view, Landy and Goldstone (2010) found that it affects performance when solving simple arithmetic problems. For instance, a spatial separation of digits congruent with the rules of arithmetic, such as 2+ 2×2 was shown to facilitate calculation performance. RTs are shorter than in incongruent trials, such as 2+2 ×2. Thus, the processing of formal operations is systematically biased by the spacing between the symbols. Namely, non-formal properties, such as spatial information, may interfere with knowledge about the order of the operations. This effect does not only hold for arithmetic. Landy and Goldstone (2007) showed that features such as similarity or connectedness affect performance in algebraic problems. Although Landy et al. (2014) interpret these results with respect to the SNA, they may also be understood with reference to general rules of perception, that is, the Gestalt law of proximity (Wertheimer, 1923) and other domain-general factors. Namely, performance in such calculation tasks can be modulated by perceptual factors (influencing visual grouping of the stimuli presented) that affect performance in classical tasks assessing cognitive control (e.g., executive attention tasks). Similar effects were previously demonstrated to influence the interference effect in the flanker task (Eriksen and Eriksen, 1974). Interference is smaller when a target stimulus and distractors (“flankers”) are spatially further apart (Miller, 1991).

Summing up, visual grouping processes that are evident in several domain-general cognitive tasks also play a considerable role in number processing at different levels, and to-be-inhibited visual stimuli are semantically processed, influencing task performance.

## Attention and conflict monitoring

Multi-digit number processing (Hinrichs et al., 1981) also depends largely on domain-general processes (Huber et al., 2016). Multi-digit integers are composed of at least two Arabic digits, arranged in place-value order. Usually processing these numbers requires focusing on digits placed in particular positions, while refraining from processing other numbers. For instance, assessing parity requires focusing only on the unit. On the other hand, comparing magnitudes requires focusing on the highest powers of 10. Only when digits in the highest power position are equal, one needs to systematically move stepwise toward the lower power positions. Thus, such tasks require an appropriate strategy for the allocation of attentional resources. Unsurprisingly, in both of these tasks, robust compatibility effects were observed (Nuerk et al., 2001, 2015). In the magnitude comparison task RTs are shorter and error rates are lower in case of unit-decade compatible number pairs, i.e., 53_68 (5 < 6 and 3 < 8). In case of incompatible pairs such as 48_63 (4 < 6 but 8 > 3) RTs increase and error rates are higher (Nuerk et al., 2001). The same is true in case of processing magnitudes of decimals, fractions, and negative numbers (Huber et al., 2015). In multi-digit number processing tasks, signatures of cognitive control processes known from classical experimental psychology are also observed. For instance, when the proportion of incongruent trials in the Stroop task decreases, interference caused by them increases (MacLeod, 1991). Analogously in the multi-digit number comparison task, as the ratio of incompatible vs. compatible trials decreases, the unit-decade compatibility effect gets stronger (Macizo and Herrera, 2013; Huber et al., 2016).

Furthermore, it was demonstrated that participants allocate their attention depending on the task conditions. When the number comparison task comprises only between-decade trials (e.g., 47_62 or 42_57; the decade numbers differ and are decisive for the decision), participants tend to focus on decade magnitude, and the effect of unit-decade compatibility is reduced or disappears totally, because the irrelevant unit causing interference is rarely attended (Huber et al., 2014). When the within-decade filler trials (i.e., trials in which the unit digit is decisive, e.g., 42_47) are introduced, the unit-decade compatibility effect increases, because participants fixate more on the units even in between-decade trials. Eye-fixation behavior and unit interference has been shown to depend on the proportion of fillers: The more often the unit digit is relevant, the larger the proportions of fixation on the unit in the experiment and the larger the unit-decade compatibility effect (Macizo and Herrera, 2011; Moeller et al., 2011; for children see Mann et al., 2011; Huber et al., 2014). Thus, the effects observed in studies on multi-digit number processing (i.e., proportion of congruent and incongruent trials as well as presence of fillers) correspond to the typical pattern of behavior in tasks assessing cognitive control (including executive attention). Both effects could be explained with reference to conflict monitoring, namely, that the participants unconsciously choose a strategy that is optimal for handling given task requirements and the overall degree of interference contained in them (Botvinick et al., 2001). On the other hand, when the distance between the unit digits of the two numbers in a number pair is large, unit-decade compatibility appears to be greater (Nuerk et al., 2001). This suggests that this effect is modulated by domain-specific processing of the multi-digit number (Bahnmueller et al., 2015).

Summing up, we can say that domain-general processes involved in allocating attention and conflict monitoring play an essential role in multi-digit number processing as well. Nevertheless, further research is needed to shed more light on the interplay between domain-general and domain-specific factors in this field.

## The role of inhibition

The previous paragraphs focused on the processing of symbolic numbers. Now effects of domain-general processing on non-symbolic magnitudes will be discussed. The non-symbolic magnitude comparison task, in which participants compare two numerosities, like sets of dots, is thought to provide a measure of the efficiency of the ANS as assessed by the internal Weber fraction (Piazza et al., 2004, 2010; Gilmore et al., 2011). The ANS is considered to be a domain-specific cognitive module to compare numerosities regardless of the visual properties of objects. In such tasks it is impossible, however, to isolate numerosities from visual properties of the stimuli (Gebuis and Reynvoet, 2012; Szucs et al., 2014). In practice, researchers try to manipulate the visual parameters across trials so that none of these parameters is necessarily linked to the number of dots. The individual trial may be congruent (numerical cue and visual parameters would be leading to the same response) or incongruent (they would lead to opposing responses). This situation leads to a Stroop-like congruency effect, which is expressed as the difference in RT and accuracy between both types of trials (Soltesz et al., 2010). As a rule, children show a larger congruency effect than adults (Szucs et al., 2007). Some explanations take recourse to domain-specific factors, such as an impaired ANS, but others to the influence of cognitive control (Szucs et al., 2013). According to the latter, the congruency effect occurs as a result of distraction by the task-irrelevant visual cue and inefficient inhibition of processing these features in incongruent trials. Thus, the larger congruency effect in children appears as a consequence of their poorer inhibition ability (Huizinga et al., 2006). Furthermore, although performance in typical ANS tasks correlates positively with children's mathematical achievement—which would provide evidence for the importance of the ANS for mathematical skills (Piazza et al., 2010)—this relation is modulated by inhibitory control (Espy et al., 2004). Gilmore et al. (2013) showed that the correlation of performance in ANS tasks and mathematical performance appears to be significant only when incongruent trials (i.e., those requiring inhibition) are considered. Developmental trajectories of refinements of number representations are to some extent congruent with developmental trajectories of improvements in cognitive control (Gilmore et al., 2013).

Summing up, it seems that the role of domain-general processes in a flagship of numerical cognition studies—the non-symbolic comparison task—has been often neglected (see Leibovich et al., 2016, for similar arguments). Executive functions seem to mediate the widely-debated relation between the non-symbolic comparison task and school math achievement.

## Conclusions

The above examples were chosen to show that different levels of domain-general processes influence numerical cognition at different levels of numerical processing. Therefore, we argue that their influence should be considered more thoroughly and systematically in the future in both correlation and experimental designs. From the examples we presented, one may get the impression that the simplest aspects of number/quantity processing are influenced by domain-general factors acting relatively late in the sequence of information processing steps, whereas more complex numerical processing is influenced by domain-general factors operating at early stages of information processing. Nevertheless, we would rather refrain from such a conclusion, and instead stress the existence of such influences across all levels both for domain-general factors and elementary number processing.

The aspects we pointed at are only notable examples to illustrate our argument in this article. One could list several other cognitive processes that are tightly related to number processing: Working memory plays a crucial role in performing complex arithmetic operations, where one needs to retrieve facts from long-term memory, conduct several operations, store interim results, and systematically update them to prevent interference (LeFevre et al., 2005). Executive functions play an important role not only in non-symbolic comparisons but correlate strongly with school math performance (Van der Ven et al., 2012). Complex reasoning, mental transformations, and ability to follow appropriate rules play a vital role in conducting mathematical proofs. Linguistic factors were also demonstrated to influence several aspects of numerical processing (see Dowker and Nuerk, 2016 for an overview). We are aware that many other influences exist. However, this opinion article is aimed at pointing out the importance of domain-general influences on different levels of number processing by choosing specific examples. A comprehensive review is beyond the scope of this opinion piece.

Summing up, we strongly emphasize that domain-general factors need to be more widely and thoroughly considered in the field of numerical cognition. In several areas, the role of domain-general factors seems to be at least as important (and sometimes apparently more important) than the role of domain-specific factors (Szucs et al., 2014). Therefore, we would like to stress that the crucial role of these processes is by no means limited to NS/ANS and as such, it must be intensely investigated along with domain-specific factors in virtually all fields within numerical cognition.

## Author contributions

MH, KC, HN, and KW reviewed the literature and developed the theoretical stance. MH and KC wrote the manuscript. MH, KC, HN, and KW reviewed and accepted its final version. The contribution of MH and KC is equal.

## Funding

MH is supported by the research grant 2015/19/B/HS1/03310 “Mechanisms of geometric cognition” funded by National Science Centre, Poland. HN and KC are supported by funding of the German Research Foundation (DFG NU 265/3-1) on “Linguistic Influences on Numerical Cognition: A cross-cultural investigation using natural specificities of Polish and German languages.”

### Conflict of interest statement

The authors declare that the research was conducted in the absence of any commercial or financial relationships that could be construed as a potential conflict of interest.

## Footnotes

^1^The term “Approximate Number System” usually refers to the ability of processing nonsymbolic numerosities (e.g., dot patterns; see e.g., Halberda et al., 2008). To the contrary, the term “Number sense” refers to the whole domain of elementary number processing (e.g., Dehaene, 2001), which apart from approximation of nonverbally presented numerosities comprises also subitizing (ability to quickly and effortlessly assess the number of elements of small sets containing less than 4 elements), understanding of the correspondence between symbolic and nonsymbolic numbers, or dot counting. Nevertheless, it should be mentioned, that the definition of the “number sense” differs vastly between authors. Berch (2005) lists 30 components of the number sense proposed by various authors. In general, when used by psychologists, the term “number sense” usually refers to more elementary processing of numerical information (especially nonsymbolic is emphasized), whereas in the field of education it also comprises more complex processes, such as arithmetic knowledge and simple calculation skills.

^2^Behavioral effects observed in tasks requiring number processing such as associating numbers with space, differences in reaction time and/or accuracy of comparing numbers / sets depending on numerical differences between stimuli to be compared.

## References

[B1] Bahnmueller J. Moeller K. Mann A. Nuerk H.-C. (2015). On the limits of language influences on numerical cognition – no inversion effects in three-digit number magnitude processing in adults. Front. Psychol. 6, 17–10. 10.3389/fpsyg.2015.01216 26322010 PMC4532912

[B2] Berch D. B. (2005). Making sense of number sense: implications for children with mathematical disabilities. J. Learn. Disabil. 38, 333–339. 10.1177/00222194050380040901 16122065

[B3] Botvinick M. M. Braver T. S. Barch D. M. Carter C. S. Cohen J. D. (2001). Conflict monitoring and cognitive control. Psychol. Rev. 108, 624–652. 10.1037/0033-295X.108.3.624 11488380

[B4] Cipora K. Hohol M. Nuerk H.-C. Willmes K. Brożek B. Kucharzyk B.. (2016). Professional mathematicians differ from controls in their spatial-numerical associations. Psychol. Res. 80, 710–726. 10.1007/s00426-015-0677-6 26063316 PMC4889706

[B5] Cipora K. Nuerk H.-C. (2013). Is the SNARC effect related to the level of mathematics? No systematic relationship observed despite more power, more repetitions, and more direct assessment of arithmetic skill. Q. J. Exp. Psychol. 66, 1974–1991. 10.1080/17470218.2013.772215 23473520

[B6] Cipora K. Patro K. Nuerk H.-C. (2015). Are spatial-numerical associations a cornerstone for arithmetic learning? The lack of genuine correlations suggests no. Mind Brain Educ. 9, 190–206. 10.1111/mbe.12093

[B7] Dehaene S. (2001). Precis of the number sense. Mind Lang. 16, 16–36. 10.1111/1468-0017.00154

[B8] Dehaene S. (2011). The Number Sense (Revised). Oxford: Oxford University Press.

[B9] De Smedt B. Noël M.-P. Gilmore C. K. Ansari D. (2013). How do symbolic and non-symbolic numerical magnitude processing skills relate to individual differences in children's mathematical skills? A review of evidence from brain and behavior. Trends Neurosci. Educ. 2, 48–55. Available online at: https://doi.org/10.1016/j.tine.2013.06.001

[B10] Desoete A. (2015). Cognitive predictors of mathematical abilities and disabilities, in The Oxford Handbook of Numerical Cognition, eds Cohen Kadosh R. Dowker A. (Oxford: Oxford University Press), 915–932.

[B11] Dowker A. Nuerk H.-C. (2016). Linguistic influences on mathematics. Front. Psychol. 7, 1–4. 10.3389/fpsyg.2016.01035 27462286 PMC4940406

[B12] Eriksen B. A. Eriksen C. W. (1974). Effects of noise letters upon the identification of a target letter in a nonsearch task. Percept. Psychophys. 16, 143–149. 10.3758/BF03203267

[B13] Espy K. A. McDiarmid M. M. Cwik M. F. Stalets M. M. Hamby A. Senn T. E. (2004). The contribution of executive functions to emergent mathematic skills in preschool children. Dev. Neuropsychol. 26, 465–486. 10.1207/s15326942dn2601_6 15276905

[B14] Feigenson L. Dehaene S. Spelke E. S. (2004). Core systems of number. Trends Cogn. Sci. 8, 307–314. 10.1016/j.tics.2004.05.002 15242690

[B15] Gebuis T. Reynvoet B. (2012). The interplay between nonsymbolic number and its continuous visual properties. J. Exp. Psychol. Gen. 141, 642–648. 10.1037/a0026218 22082115

[B16] Gilmore C. K. Attridge N. Clayton S. Cragg L. Johnson S. Marlow N. (2013). Individual differences in inhibitory control, not non-verbal number acuity, correlate with mathematics achievement. PLoS ONE 8:e67374 10.1371/journal.pone.0067374 23785521 PMC3681957

[B17] Gilmore C. K. Attridge N. Inglis M. (2011). Measuring the approximate number system. Q. J. Exp. Psychol. 64, 2099–2109. 10.1080/17470218.2011.574710 21846265

[B18] Halberda J. Mazzocco M. M. Feigenson L. (2008). Individual differences in non-verbal number acuity correlate with maths achievement. Nature 455, 665–668. 10.1038/nature07246 18776888

[B19] Hinrichs J. V. Yurko D. S. Hu J.-M. (1981). Two-digit number comparison: use of place information. J. Exp. Psychol. Hum. Percept. Perform. 7, 890–901. 10.1037/0096-1523.7.4.890

[B20] Huber S. Cornelsen S. Moeller K. Nuerk H.-C. (2015). Toward a model framework of generalized parallel componential processing of multi-symbol numbers. J. Exp. Psychol. Learn. Mem. Cogn. 41, 732–745. 10.1037/xlm0000043 25068853

[B21] Huber S. Mann A. Nuerk H.-C. Moeller K. (2014). Cognitive control in number magnitude processing: evidence from eye-tracking. Psychol. Res. 78, 539–548. 10.1007/s00426-013-0504-x 23877614

[B22] Huber S. Nuerk H.-C. Willmes K. Moeller K. (2016). A general model framework for multi-symbol number comparison. Psychol. Rev. 123, 667–695. Available online at: https://doi.org/10.1037/rev0000040 27617347 10.1037/rev0000040

[B23] Huizinga M. Dolan C. V. van der Molen M. W. (2006). Age-related change in executive function: developmental trends and a latent variable analysis. Neuropsychologia 44, 2017–2036. 10.1016/j.neuropsychologia.2006.01.010 16527316

[B24] Landy D. Allen C. Zednik C. (2014). A perceptual account of symbolic reasoning. Front. Psychol. 5:275. 10.3389/fpsyg.2014.00275 24795662 PMC4001060

[B25] Landy D. Goldstone R. L. (2007). How abstract is symbolic thought? J. Exp. Psychol. Learn. Mem. Cogn. 33, 720–733. 10.1037/0278-7393.33.4.720 17576149

[B26] Landy D. Goldstone R. L. (2010). Proximity and precedence in arithmetic. Q. J. Exp. Psychol. 63, 1953–1968. 10.1080/17470211003787619 20509096

[B27] LeFevre J.-A. DeStefano D. Coleman B. Shanahan T. (2005). Mathematical cognition and working memory, in Handbook of Mathematical Cognition, ed Campbell J. I. D. (New York, NY: Psychology Press), 361–377.

[B28] Leibovich T. Ansari D. (2016). The symbol-grounding problem in numerical cognition: a review of theory, evidence, and outstanding questions. Can. J. Exp. Psychol. 70, 12–23. 10.1037/cep0000070 26913782

[B29] Leibovich T. Katzin N. Harel M. Henik A. (2016). From ‘sense of number’ to ‘sense of magnitude’ – The role of continuous magnitudes in numerical cognition. Behav. Brain Sci. 10.1017/S0140525X16000960. [Epub ahead of print]. 27530053

[B30] Lyons I. M. Nuerk H.-C. Ansari D. (2015). Rethinking the implications of numerical ratio effects for understanding the development of representational precision and numerical processing across formats. J. Exp. Psychol. Gen. 144, 1021–1035. 10.1037/xge0000094 26168037

[B31] Macizo P. Herrera A. (2011). Cognitive control in number processing: evidence from the unit–decade compatibility effect. Acta Psychol. 136, 112–118. 10.1016/j.actpsy.2010.10.008 21078509

[B32] Macizo P. Herrera A. (2013). The processing of Arabic numbers is under cognitive control. Psychol. Res. 77, 651–658. 10.1007/s00426-012-0456-6 23053596

[B33] MacLeod C. M. (1991). Half a century of research on the Stroop effect: an integrative review. Psychol. Bull. 109, 163–203. 10.1037/0033-2909.109.2.163 2034749

[B34] Mann A. Moeller K. Pixner S. Kaufmann L. Nuerk H.-C. (2011). Attentional strategies in place-value integration. Zeitschrift für Psychol. 219, 42–49. 10.1027/2151-2604/a000045

[B35] Miller J. (1991). The flanker compatibility effect as a function of visual angle, attentional focus, visual transients, and perceptual load: a search for boundary conditions. Percept. Psychophys. 49, 270–288. 10.3758/BF03214311 2011464

[B36] Moeller K. Huber S. Nuerk H.-C. Willmes K. (2011). Two-digit number processing: holistic, decomposed or hybrid? A computational modelling approach. Psychol. Res. 75, 290–306. 10.1007/s00426-010-0307-2 20798955

[B37] Nuerk H.-C. Moeller K. Willmes K. (2015). Multi-digit number processing: overview, conceptual clarifications, and language influences, in The Oxford Handbook of Numerical Cognition, eds Cohen Kadosh R. Dowker A. (Oxford: Oxford University Press), 106–139.

[B38] Nuerk H.-C. Weger U. Willmes K. (2001). Decade breaks in the mental number line? Putting the tens and units back in different bins. Cognition 82, B25–B33. 10.1016/S0010-0277(01)00142-1 11672709

[B39] Piazza M. Facoetti A. Trussardi A. N. Berteletti I. Conte S. Lucangeli D.. (2010). Developmental trajectory of number acuity reveals a severe impairment in developmental dyscalculia. Cognition 116, 33–41. 10.1016/j.cognition.2010.03.012 20381023

[B40] Piazza M. Izard V. Pinel P. Le Bihan D. Dehaene S. (2004). Tuning curves for approximate numerosity in the human intraparietal sulcus. Neuron 44, 547–555. 10.1016/j.neuron.2004.10.014 15504333

[B41] Rousselle L. Palmers E. Noël M.-P. (2004). Magnitude comparison in preschoolers: what counts? Influence of perceptual variables. J. Exp. Child Psychol. 87, 57–84. 10.1016/j.jecp.2003.10.005 14698689

[B42] Soltesz F. Szucs D. Szucs L. (2010). Relationships between magnitude representation, counting and memory in 4- to 7-year-old children: a developmental study. Behav. Brain Funct. 6:13. 10.1186/1744-9081-6-13 20167066 PMC2833140

[B43] Szucs D. Devine A. Soltesz F. Nobes A. Gabriel F. (2014). Cognitive components of a mathematical processing network in 9-year-old children. Dev. Sci. 17, 506–524. 10.1111/desc.12144 25089322 PMC4253132

[B44] Szucs D. Nobes A. Devine A. Gabriel F. C. Gebuis T. (2013). Visual stimulus parameters seriously compromise the measurement of approximate number system acuity and comparative effects between adults and children. Front. Psychol. 4, 1–13. 10.3389/fpsyg.2013.00444 23882245 PMC3715731

[B45] Szucs D. Soltész F. Jármi É. Csépe V. (2007). The speed of magnitude processing and executive functions in controlled and automatic number comparison in children: an electro-encephalography study. Behav. Brain Funct. 3, 23–20. 10.1186/1744-9081-3-23 17470279 PMC1872027

[B46] Van der Ven S. H. G. Kroesbergen E. H. Boom J. Leseman P. P. M. (2012). The development of executive functions and early mathematics: a dynamic relationship. Br. J. Educ. Psychol. 82, 100–119. 10.1111/j.2044-8279.2011.02035.x 22429060

[B47] Wertheimer M. (1923). Untersuchungen zur Lehre von der Gestalt. II. Psychol. Res. 4, 301–350. 10.1007/BF00410640

